# Sinularin induces DNA damage, G2/M phase arrest, and apoptosis in human hepatocellular carcinoma cells

**DOI:** 10.1186/s12906-017-1583-9

**Published:** 2017-01-19

**Authors:** Ting-Wen Chung, Shih-Chao Lin, Jui-Hsin Su, Yu-Kuo Chen, Chi-Chien Lin, Hong-Lin Chan

**Affiliations:** 1 Institute of Bioinformatics and Structural Biology and Department of Medical Sciences, National Tsing Hua University, Hsinchu, Taiwan; 2 Ph.D. Program in Medical Biotechnology, National Chung Hsing University, Taichung, Taiwan; 3 SRI international, Harrisonburg, VA USA; 4 Taiwan Coral Research Center, National Museum of Marine Biology & Aquarium, Pingtung, Taiwan; 5 Department of Food Science, National Pingtung University of Science and Technology, Pingtung, Taiwan; 6 Institute of Biomedical Science, National Chung-Hsing University, Taichung, Taiwan; 7 Department of Biotechnology, Asia University, Taichung, Taiwan; 8 Department of Medical Research, China Medical University Hospital, Taichung, Taiwan

**Keywords:** Sinularin, *Sinularia flexibilis*, Hepatocellular carcinoma, G2/M, Apoptosis, DNA damage

## Abstract

**Background:**

Sinularin isolated from the cultured soft coral *Sinularia flexibilis* has been reported to exert potent cytotoxic effects against particular types of cancer. This study was carried out to investigate the cytotoxic effects in sinularin-treated human hepatocellular carcinoma cells, HepG2, and to subsequently explore the underlying molecular mechanisms.

**Methods:**

TheMTT (3-[4,5-dimethylthiazol-2-yl]-2, 5-diphenyl- tetrazolium bromide) method was used to evaluate the cytotoxicity of sinularin on HepG2 and Hep3B cell lines. Furthermore, the cell cycle distribution assay, apoptosis assay, and western blot analysis in vitro were used to explore the possible mechanisms of action.

**Results:**

From the results of our study, cell viability was obviously inhibited by sinularin in a dose-dependent manner. In addition, our results suggested that sinularin triggered DNA damage and subsequently induced cell cycle G2/M arrest associated with up-regulation of p-ATM (Ser(1981)), p-Chk2 (Tyr(68)), p-cdc2 (Tyr(15)), and p53 coupled with increased expression of downstream proteins p21 and down-regulation of p-cdc25 (Ser(216)). Moreover, the results of the apoptosis assay and western blot analysis indicated that the cytotoxic activity could be related to mitochondrial apoptosis, characterized by decrease of Bcl-2 expression, disruption of mitochondrial membrane potential, and sequential activation of caspases and Poly (ADP-ribose) polymerase (PARP).

**Conclusions:**

This study reveals for the first time the anti-HCC activities of sinularin, the active compound isolated from the cultured soft coral *Sinularia flexibilis*. We believe that our results warrant further evaluation of sinularin as a new anti-HCC chemotherapeutic agent.

## Background

Hepatocellular carcinoma (HCC) is a primary liver malignancy and the second highest cause of cancer-related death in the world [1]. Without specific treatment, HCC carries a dismal prognosis and the reported 5-year survival rate is as low as approximately 10% [2]. The incidence of HCC is highly related to endemic hepatitis viral infections such as HBV, HCV, or HDV. Currently, there are only a few strategies for HCC treatment – radical treatments such as surgical resection and orthotopic liver transplantation (OLT), radiation therapy, and chemotherapy. However, most HCC patients’ liver cancers are in middle to advanced stages upon diagnosis. As a result, they cannot be selected as candidates for these radical treatments. Thus, anticancer drugs are a more feasible option for treating advanced HCC. Sorafenib, a serine-threonine kinase inhibitor, is currently the only approved systematic drug for anti-HCC. Unfortunately, the efficacy of sorafenib is limited, and some patients are intolerant [3]. Therefore, more effective therapeutic strategies for HCC are needed.

Sinularin is a natural product isolated from cultured soft coral, *Sinularia flexibilis*, and since 1977 has been shown to possess antineoplastic activity against human epidermoid carcinoma and P388 lymphocytic leukemia [4]. Despite its significant anti-cancer activities, very few studies have focused on sinularin research. To date, two studies reported that sinularin exhibited anti-tumor activities in human gastric carcinoma and melanoma cells [5, 6]. In addition to anti-tumor effects, sinularin has been shown to inhibit the production of inflammatory mediators such as inducible nitric oxide synthase (iNOS) and cyclooxygenase-2 (COX-2) stimulated by lipopolysaccharide (LPS) [7]. As a result, sinularin is a bioactive compound with multiple functions in tumor suppression and immunomodulation.

The mechanisms of anti-cancer activity exerted by sinularin are not clear and require further investigation. To our knowledge, there are currently no scientific reports focused on the antitumor effects of sinularin against human liver cancer cells. Thus, in this study, we utilized sinularin to evaluate its cytotoxic effects on human hepatocellular carcinoma cells and investigate the mechanistic pathways of cancer cell death.

## Methods

### Chemicals

The marine natural compound, sinularin, was isolated from the soft coral *S. flexibilis* as described [5] and kindly provided by Dr. Jui-Hsin Su (National Museum of Marine Biology & Aquarium, Pingtung, Taiwan); sorafenib was purchased from Cayman Chemical (Ann Arbor, MI USA). Sinularin and sorafenib were dissolved in dimethyl sulfoxide (DMSO; Sigma-Aldrich, St. Louis, MO USA) at 100 mM as stock solution for in vitro assays. Henceforth, DMSO and sorafenib were used as vehicle control and positive control, respectively.

### Cell lines and culture

The human hepatocellular carcinoma cell lines, HepG2 and Hep3B, were purchased from American Type Culture Collection (ATCC^®^) (Manassas, VA USA). The growth medium is DMEM with 10% (v/v) fetal bovine serum (FBS), streptomycin (100 μg/mL) and penicillin (100 U/ml) (Gibco/BRL, Gran Island, NY USA). Cells were cultured in incubators with 5% CO_2_ at 37 °C.

### MTT assay

Cell viability was monitored by the MTT colorimetric assay (BioVision, Milpitas, CA USA). Briefly, 200 μL of MTT (0.5 mg/mL) was added to each well and incubated for 4 h at 37 °C after removing the culture supernatants. MTT crystals were dissolved by DMSO and the absorbance was measured at 540 nm wavelength with an ELISA microplate reader (Tecan Sunrise, San Jose, CA USA). All MTT assays were performed in 96-well plates and repeated at least three times.

### Cell cycle assay

Sinularin- or sorafenib- treated cells were harvested, washed with PBS, and fixed with 70% ethanol at −20 °C overnight. The fixed cells were stained by propidium iodide (PI; Sigma-Aldrich, St. Louis, MO USA) containing RNase A (Sigma-Aldrich, St. Louis, MO USA) for 30 min in the dark at room temperature. The cells were then analyzed by an Accuri^TM^ C5 cytometer (BD Biosciences, San Jose, CA USA). Data were further analyzed by C6 Accuri system software (BD Biosciences, San Jose, CA USA).

### Annexin V-FITC/PI staining assay

The Annexin V-FITC/PI staining assay was performed using the FITC Annexin V Apoptosis Detection Kit (BioVision, Milpitas, CA USA) in accordance with manufacturer’s instructions. In brief, sinularin-treated cells were trypsinized and gently washed with cold PBS twice followed by resuspension in binding buffer. Equal volumes of Annexin V-FITC and propidium iodide were added to stain cells for 10 min at room temperature in the dark prior to determining the percentage of apoptotic cells with an Accuri^TM^ C5 cytometer.

### Measurement of mitochondrial membrane potential (MMP)

HepG2 cells treated with sinularin for 24 h were trypsinized, washed with PBS, and resuspended with culture medium followed by staining with JC-1 solution (10 ng/μL) (Life Invitrogen, Carlsbad, CA USA) for 10 min at 37 °C. Changes in mitochondrial membrane potential were then examined by an Accuri^TM^ C5 cytometer.

### Western blotting

Cells were harvested with lysis buffer after treatments at the indicated time points. BCA Protein Assay Reagent (Thermo Fisher Scientific, Waltham, MA USA) was utilized to measure the protein concentrations before performing SDS-polyacrylamide gel electrophoresis. Proteins were then transferred to PVDF membranes for subsequent antibody hybridization. The membranes were blocked with 5% non-fat milk at room temperature for 1 h and blotted with primary antibody at 4 °C for overnight incubation, followed by secondary antibodies conjugated with horseradish peroxidase (HRP) (Jackson Laboratory, Bar Harbor, ME USA) at 4 °C overnight. The immunoactive bands were detected with an enhanced chemiluminescence (ECL) system and developed using the LAS3000 system (Fujifilm, Valhalla, NY USA). In this study, the primary antibodies obtained from Cell Signaling Technology Inc. (Beverly, MA USA) were anti-phospho-cdc25-ser-216, anti-cdc25, anti-cyclin B1, anti-p53, anti-p21, anti-cleaved-caspase-3, anti-BCL-2, anti-cleaved-caspase-8, anti-cleaved-caspase-9, anti-cleaved-PARP, anti-Bax, anti-phospho-ATM-ser-1981, anti-ATM, anti-CHK-2, anti-ATR-ser-428, anti-ATR anti-phospho-CHK-2-Thr-68, anti-phospho-CHK-1-ser-345, anti-CHK-1 antibodies, from Epitomics (Burlingame, CA USA) were anti-cdc2-Tyr-15 and anti-cdc2 antibodies, from GeneTex (Sann Antonio, TX USA) were anti-cyclin B1 and anti-GAPDH antibodies, and from Millpore (Billerica, MA USA) were anti-p-H2A.X and anti-GAPDH antibodies.

### Statistical analysis

The results were expressed as the mean ± SD. All statistical analyses were performed with the GraphPad Prism software package version 5.0. Experimental data grouped by one variable were evaluated by one-way analysis of variable followed by Tukey’s test. Statistical significance is considered if *P* value is less than 0.05 (*P* < 0.05).

## Results

### Sinularin reduced the viability of human liver cancer cells

**Fig. 1:**
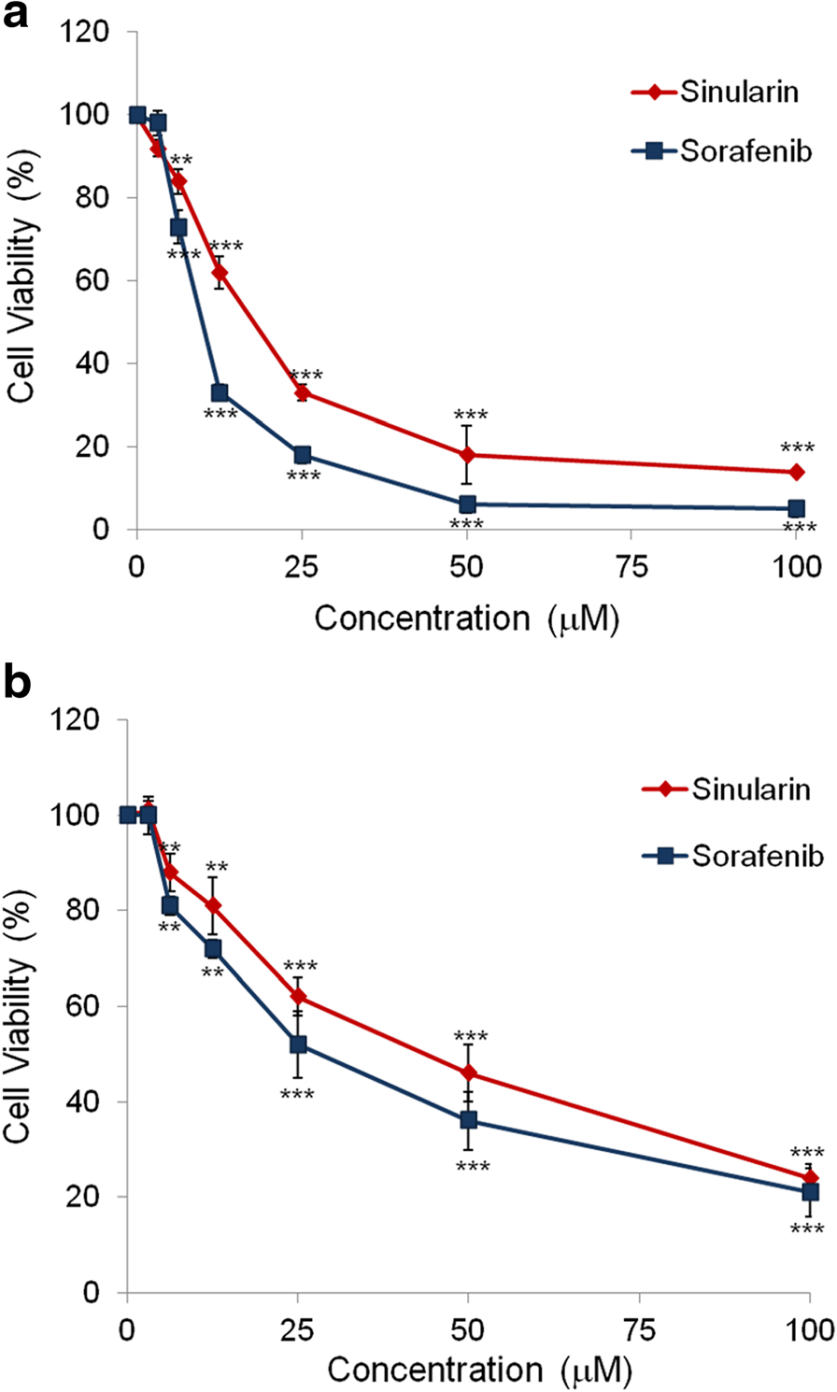
Analysis of cell viabilities in HCC cell lines. Percentages of cell viabilities of (**a**) HepG2 and (**b**) Hep3B cells after treating with sinularin or sorafenib at different concentrations for 24 h. Cell viabilities were plotted and represented as means ± SD. All data presented are representatives of three independent experiments with similar results. Significant differences from DMSO treated control group are indicated by **p < 0.01, ***p < 0.001

The cytotoxic effect of sinularin on the viability of liver cancer cell lines was determined by MTT assay. We treated two human liver cancer cell lines (HepG2 and Hep3B) with sinularin or sorafenib, a positive control, at different concentrations for 24 h. As shown in Fig. 1, both sinularin and sorafenib induced cytotoxicity in human liver cancer cell lines in a dose-dependent manner. Notably, these two liver cell lines exhibited different sensitivities to sinularin and sorafenib; the inhibitory concentration 50% (IC_50_) values of sinularin and sorafenib to HepG2 were 17.5 ± 6.7 μM and 9.4 ± 2.3 μM respectively, while the IC_50_ values to Hep3B were 43.2 ± 8.1 μM and 33.9 ± 8.6 μM respectively. The results indicated that sinularin has a similar cytotoxic effect as sorafenib on both HepG2 and Hep3B. Also, the HepG2 cell line was highly sensitive to sinularin treatments; thus, we selected HepG2 to perform further analysis and evaluation of the cytotoxic potency of sinularin.

### Sinularin induced G2/M arrest and apoptosis in HepG2 cells

**Fig. 2:**
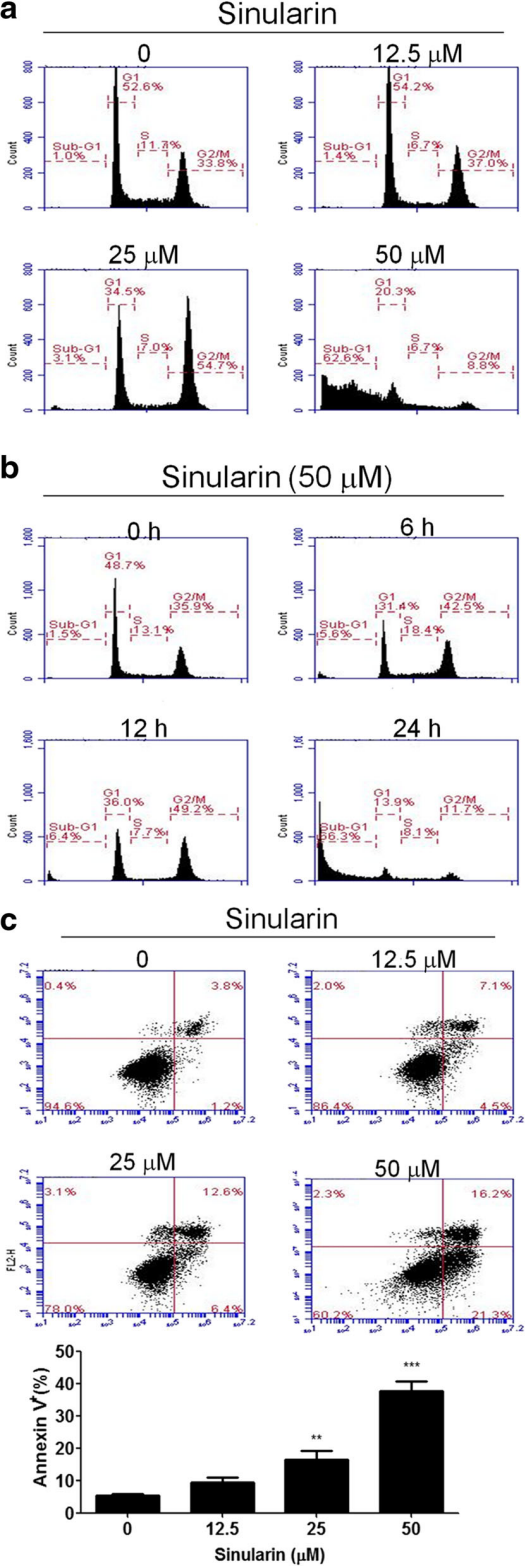
Cell cycle distributions of HepG2 cells with sinularin treatments. **a** Cell cycle analysis of sinularin-treated cells. HepG2 cells were treated with the indicated concentrations of sinularin for 24 h, or (**b**) treated with 50 μM of sinularin for 6, 12, and 24 h. Cell cycle distributions were determined by propidium iodide (PI) staining and flow cytometry analysis. Data are representative of three independent experiments with similar results. **c** Analysis of sinularin-induced apoptosis in HepG2 cells at indicated concentrations for 24 h. Phosphatidylserine externalization and DNA integrity were determined by FITC-annexin-V and PI, respectively. The lower-right quadrant (annexin-V^+^/PI^−^) represents early apoptosis, while the upper-right quadrant (annexin V^+^/PI^+^) indicates late apoptosis and necrosis. The means ± SD of the experimental triplicates are presented in the bar graph at the bottom. All data presented are representatives of three independent experiments with similar results. Significant differences from DMSO treated control group are indicated by **p < 0.01, ***p < 0.001

To further understand the functional mechanisms of sinularin in inhibiting cell growth, we studied the cell cycle profiles of HepG2 cells after sinularin treatment. Fig. 2a shows that 24-h treatment with sinularin caused high populations of HepG2 cells arrested in G2/M checkpoint at 25 μM concentrations. The accumulation of the sub-G1 phase population at 50 μM group indicated that high doses of sinularin led to massive cell death within 24 h. We subsequently analyzed the cell cycle profiles of HepG2 cells treated with 50 μM sinularin at 6, 12, and 24 h and found that high concentrations of sinularin induced significant accumulation of HepG2 cells in the G2/M phase after 6- and 12-h treatments (Fig. 2b); thus, the sub-G1 phase population increased at 24 h post-treatment.

We further tested whether sinularin elicited apoptotic cell death in HepG2 cells at 24 h of sinularin treatment by staining phosphatidylserine with annexin V conjugated with fluorescein isothiocyanate (FITC) and DNA with propidium iodide (PI). As shown in Fig. 2c, the percentages of early apoptotic (annexin V^+^/PI^−^ in lower-right quadrants) and late apoptotic or necrotic HepG2 cells (annexin V^+^/PI^+^ in upper- right quadrants) were higher in a dose-dependent manner compared to the DMSO control, suggesting that sinularin increased cell death and caused the G2/M arrest in HepG2 cells.

### Sinularin affected the expressions of G2/M corresponding proteins in HepG2 cells

**Fig. 3:**
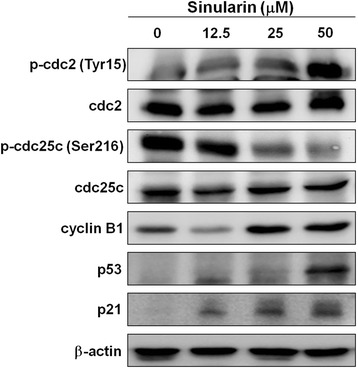
Sinularin affected the cell cycle regulatory proteins in HePG2 cells. HepG2 cells were treated with different dosages of sinularin for 12 h and western blotting was performed to quantify the expression levels of cell cycle regulatory proteins. GAPDH expressions were measured as internal controls to show equal protein loading. The data presented are representatives of three independent experiments with similar results

Next, we examined the G2/M-related protein expressions involved in cell cycle progression following sinularin treatment. The results showed that the levels of p53, and p21 increased remarkably after 12-h treatment with sinularin. Of note, the expression of phospho-Cdc25C (Ser-216) was dose-dependently suppressed, whereas phospho-Cdc2 (Tyr-15) was positively augmented at the same time point (Fig. 3). These data strongly indicated that sinularin could induce G2/M arrest by altering the expressions of G2/M corresponding proteins.

### Sinularin altered mitochondrial membrane potential (Δψ_m_)

**Fig. 4:**
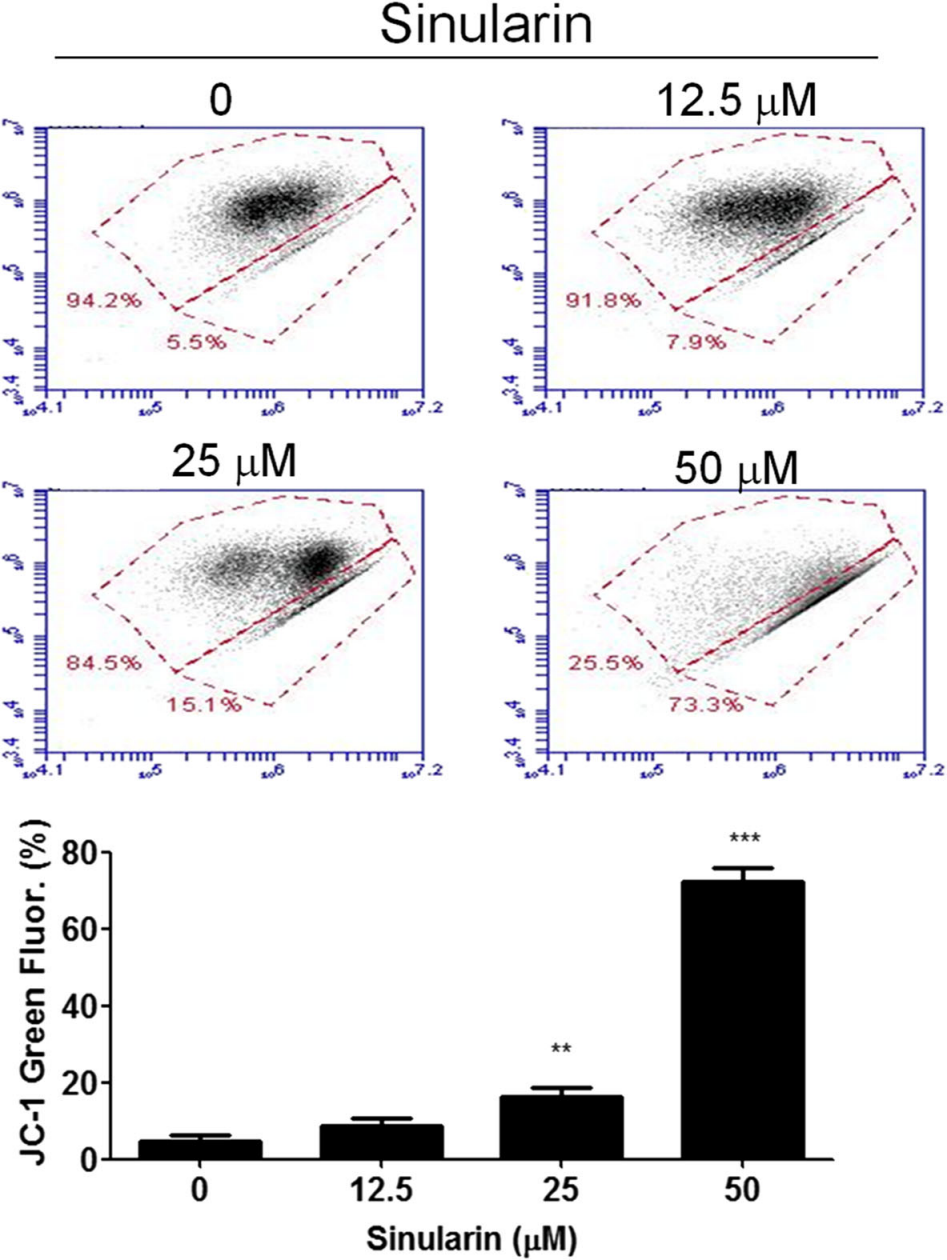
Sinularin induced MMP reduction in HepG2 cells. HepG2 cells were treated with different dosages of sinularin for 24 h. Mitochondrial membrane potential (Δψm) was determined by JC-1 fluorescent dye staining and flow cytometry analysis. The means ± SD of the experimental triplicates were presented in the bar graph at the bottom. All data presented are representatives of three independent experiments with similar results. Significant differences from DMSO treated control group are indicated by **p < 0.01, ***p < 0.001

Changes of mitochondrial membrane potential are considered indicators of cellular insults or stressors. Mitochondrial membrane potentials in apoptotic cells are low due to depolarization which can be quantified by cationic fluorescent dyes such as DiOC_6_ or JC-1 [8, 9]. Here we utilized JC-1 to measure the mitochondrial membrane potential by flow cytometry after sinularin treatment. As shown in Fig. 4, green fluorescent signals increased in HepG2 cells after sinularin treatment in a concentration-dependent manner after 24 h, suggesting that treatment with sinularin led to apoptosis in HepG2 cells and that mitochondrial dysfunction could be involved in sinularin-induced apoptosis.

### Sinularin regulated the expressions of apoptotic proteins in HepG2 cells

**Fig. 5:**
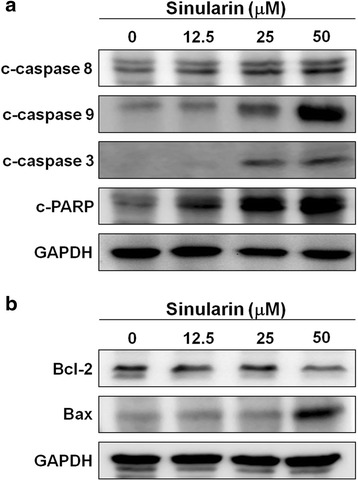
Sinularin regulated the protein levels of apoptosis in HepG2 cells. **a** and **b** Cell lysates of HepG2 cells were collected after sinularin treatments at 12.5, 25, and 50 μM for 24 h and western blotting was used to determine the protein expression levels. GAPDH expressions were measured as internal controls to show equal protein loading. The data presented are representatives of three independent experiments with similar results

The mitochondrial apoptotic pathway involves the activation of apoptosome-associated initiator caspases, such as caspases 8 and 9. Initiator caspases, in turn, activate downstream caspase 3, which subsequently induces cleavage of Poly (ADP-ribose) polymerase (PARP) [10–12]. Thus, we performed western blotting to elucidate the relationships among the apoptotic proteins in HepG2 cells after 24 h of exposure to sinularin. The data clearly demonstrated that sinularin treatment up-regulated the expressions of cleaved caspases 8, 9, 3, and PAPR (Fig. 5a). Besides, proteins in the B-cell lymphoma-2 (Bcl-2) family play distinguished roles in apoptosis. For example, Bcl-2 inhibits apoptosis whereas Bax has opposite function [13, 14]. We examined the protein expressions of Bcl-2 and Bax and found that Bcl-2 expression levels were decreased, whereas Bax was increased after sinularin treatment (Fig. 5b). These data revealed that sinularin induced apoptosis in HepG2 cells by regulating the apoptotic proteins associated with mitochondrial pathway.

### Sinularin activated ATM/Chk2-medicated DNA damages

**Fig. 6:**
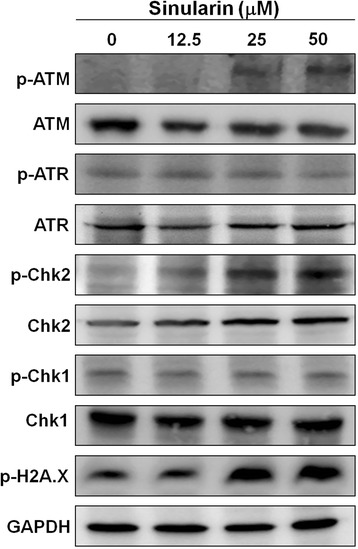
Sinularin triggered DNA damage responses in HepG2 cells. HepG2 cells were treated with sinularin at 12.5, 25, and 50 μM for 24 h followed by western blotting to measure the DNA damage related proteins, phospho and non-phospho ATM, ATR, Chk1, Chk2, and H2A.X. GAPDH expressions were measured as internal controls to show equal protein loading. The data presented are representatives of three independent experiments with similar results

We investigated whether sinularin caused DNA damage in HepG2 cells, which would in turn initiate the apoptosis pathway to achieve its anti-tumor activity. We analyzed the expression levels of DNA damage signaling molecules, including ataxia telangiectasia mutated (ATM), ATM and Rad3-related (ATR), checkpoint kinase 1 and 2 (Chk1/2), and phosphorylated histone H2A.X (p-H2A.X). As shown in Fig. 6, the levels of phosphorylated ATM on Ser-1981 and phosphorylated Chk2 on Thr-68, a downstream target of ATM, along with p-H2A.X, were significantly increased in a concentration-dependent manner after exposure to sinularin but not phospho-ATR (Ser-428) or phospho-Chk1 (Ser-345). These findings suggest that the induction of DNA damage by sinularin caused cytotoxicity in HepG2 cells.

## Discussion

Cytotoxic chemotherapy has been a mainstay treatment for advanced HCC and the chemotherapeutic agents trigger apoptosis in HCC by inducing massive DNA damage, which fail to undergo productive DNA repair [15]. It is also well-known that HCC exhibits resistance to chemotherapy or radiotherapy and recent studies have revealed that the emerging resistance of HCC to conventional therapies resulted from the induction of resistance-associated protein 2 and anti-apoptotic Bcl-2 [16, 17]. Furthermore, HCC’s resistance to multiple drugs greatly diminishes the effectiveness of chemotherapy treatments. As a result, it is necessary to develop second line chemotherapeutic agents. Although the apoptosis mechanisms induced by sinularin require further investigation, we clearly demonstrated in this study that sinularin caused PARP-mediated apoptosis of HCC via activation of cascade caspases such as caspase 3, 8, and 9 as well as by repressing Bcl-2 expression and activating Bax, which is subsequently down-regulated in HCC (Fig. 5).

DNA damage induced by anti-cancer drugs can initiate p53-dependent and p53-independent pathways to block cells at the G2/M checkpoint and from entry into mitosis by which drugs can exert their anti-cancer effects [18]. For the p53-depedent pathway, several transcriptional targets of p53, including p21 can down-regulate the expression of cdc2 and cyclin B1 in addition to directly inactivating cdc2 protein. Hence, inactive cdc2/cyclin B1 can’t further activate cdc25 which is essential for mitosis [19]. On the other hand, the p53-independent pathway of G2/M arrest is involved with DNA repair proteins such as the kinases ATM and ATR, which can activate downstream Chk1 and Chk2 kinase, respectively, and cause cdc25 to anchor in the cytoplasm [20–22]. Sinularin, in this study, was shown to not only elevate p53 and p21 expression levels (p53-dependent) but also to increase the protein levels of phosphorylated ATM/Chk2 (p53-indenpent), but not Chk1. Our results indicated that sinularin induced the DNA damage and caused G2/M arrest in HCC via both pathways. These results also explained the reason why sinularin seems to exhibit higher cytotoxicity to HepG2 with wild-type p53 than to Hep3B cells, which is a p53 null cell line (Fig. 1).

Given DNA damage usually comes along with oxidative stress and that reactive oxygen species (ROS) could be involved in leading to the mitochondria outer membrane permeabilization (MOMP) [23, 24], it is possible that sinularin induced oxidative DNA damage as well. We measured the ROS levels by using the fluorescent probe DCFDA after sinularin treatment with HCC, but there were no significant changes observed between sinularin-treated cells and untreated cells, suggesting that sinularin-induced DNA damage and cytotoxicity is not associated with the generation of ROS in human HepG2 cells (data not shown).

Sorafenib, a multiple kinase inhibitor, is the only approved chemotherapeutic agent for treating advanced HCC. Sorafenib exhibits its tumor suppression effects in cell growth, angiogenesis, and can be applied toward macrovascular invasion and extra hepatic metastasis [25]. A recent study showed that sorafenib induced more apoptosis in HepG2 than Hep3B cells due to higher expression levels of microRNA, miR-181a, in Hep3B cells, suggesting miR-181a contributes to the drug resistance of HCC [26]. In response to the anticancer drug-resistance of HCC, some second-line anticancer drugs have been evaluated in combination with sorafenib treatment. For example, an epidermal growth factor receptor (EGFR)-related tyrosine kinase enzyme inhibitor, Erlotinib, was applied for treatment of advanced HCC in a phase-III trial [27]. In this study, not only did we provide a new respect for cytotoxic chemotherapeutic strategies by applying a natural marine compound in treating HCC, but out data also revealed that the targets of sinularin are distinguished from sorafenib which makes sinularin a good candidate as a second-line antineoplastic drug.

## Conclusions

In our study, we have shown that sinularin can effectively inhibit the proliferation of human hepatocellular carcinoma HepG2 cells by increasing G2/M cell cycle arrest, inducing apoptosis, and activating DNA damage responses, thus contributing to the antitumor activity of sinularin. Overall, our results demonstrated that sinularin might be a potential candidate in HCC chemoprevention and chemotherapy.

## Abbreviations

ATM: Ataxia telangiectasia mutated
ATR: (ATM and Rad3-related)
Bcl-2: B-cell lymphoma-2
Chk: Checkpoint kinases
HCC: Hepatocellular carcinoma
PARP: Poly (ADP-ribose) polymerase
ROS: Reactive oxygen species

## References

[1] Torre LA, Bray F, Siegel RL, Ferlay J, Lortet-Tieulent J, Jemal A (2015). Global cancer statistics, 2012. CA Cancer J Clin.

[2] Altekruse SF, McGlynn KA, Reichman ME (2009). Hepatocellular carcinoma incidence, mortality, and survival trends in the United States from 1975 to 2005. J Clin Oncol.

[3] Moriguchi M, Umemura A, Itoh Y (2016). Current status and future prospects of chemotherapy for advanced hepatocellular carcinoma. Clin J Gastroenterol.

[4] Weinheimer AJ, Matson AJ, Hossain MB, van der Helm D (1977). Marine anticancer agents: sinularin and dihydrosinularin, new cembranolides from the soft coral, sinularia flexibilis. Tetrahedron Lett.

[5] Su TR, Lin JJ, Chiu CC, Chen JY, Su JH, Cheng ZJ (2012). Proteomic investigation of anti-tumor activities exerted by sinularin against A2058 melanoma cells. Electrophoresis.

[6] Wu YJ, Wong BS, Yea SH, Lu CI, Weng SH. Sinularin Induces Apoptosis through Mitochondria Dysfunction and Inactivation of the pI3K/Akt/mTOR Pathway in Gastric Carcinoma Cells. Mar Drugs. 2016;14(8):E142. 10.3390/md14080142 PMC4999903 27472346

[7] Huang SY, Chen NF, Chen WF, Hung HC, Lee HP, Lin YY (2012). Sinularin from indigenous soft coral attenuates nociceptive responses and spinal neuroinflammation in carrageenan-induced inflammatory rat model. Mar Drugs.

[8] Perry SW, Norman JP, Barbieri J, Brown EB, Gelbard HA (2011). Mitochondrial membrane potential probes and the proton gradient: a practical usage guide. Biotechniques.

[9] Rottenberg H, Wu S (1998). Quantitative assay by flow cytometry of the mitochondrial membrane potential in intact cells. Biochim Biophys Acta.

[10] Baliga B, Kumar S (2003). Apaf-1/cytochrome c apoptosome: an essential initiator of caspase activation or just a sideshow?. Cell Death Differ.

[11] Boulares AH, Yakovlev AG, Ivanova V, Stoica BA, Wang G, Iyer S (1999). Role of poly(ADP-ribose) polymerase (PARP) cleavage in apoptosis. Caspase 3-resistant PARP mutant increases rates of apoptosis in transfected cells. J Biol Chem.

[12] Chandra D, Choy G, Deng X, Bhatia B, Daniel P, Tang DG (2004). Association of active caspase 8 with the mitochondrial membrane during apoptosis: potential roles in cleaving BAP31 and caspase 3 and mediating mitochondrion-endoplasmic reticulum cross talk in etoposide-induced cell death. Mol Cell Biol.

[13] Marsden VS, O’Connor L, O’Reilly LA, Silke J, Metcalf D, Ekert PG (2002). Apoptosis initiated by Bcl-2-regulated caspase activation independently of the cytochrome c/Apaf-1/caspase-9 apoptosome. Nature.

[14] Youle RJ, Strasser A (2008). The BCL-2 protein family: opposing activities that mediate cell death. Nat Rev Mol Cell Biol.

[15] Riaz M, Zia-Ul-Haq M, Saad B (2016). Anthocyanins effects on carcinogenesis, immune system and the central nervous system, anthocyanins and human health: biomolecular and therapeutic aspects.

[16] Chun E, Lee KY (2004). Bcl-2 and Bcl-xL are important for the induction of paclitaxel resistance in human hepatocellular carcinoma cells. Biochem Biophys Res Commun.

[17] Rigalli JP, Ciriaci N, Arias A, Ceballos MP, Villanueva SS, Luquita MG (2015). Regulation of multidrug resistance proteins by genistein in a hepatocarcinoma cell line: impact on sorafenib cytotoxicity. PLoS One.

[18] Taylor WR, Stark GR (2001). Regulation of the G2/M transition by p53. Oncogene.

[19] Izumi T, Maller JL (1993). Elimination of cdc2 phosphorylation sites in the cdc25 phosphatase blocks initiation of M-phase. Mol Biol Cell.

[20] Sun SY, Hail N, Lotan R (2004). Apoptosis as a novel target for cancer chemoprevention. J Natl Cancer Inst.

[21] Uto K, Inoue D, Shimuta K, Nakajo N, Sagata N (2004). Chk1, but not Chk2, inhibits Cdc25 phosphatases by a novel common mechanism. EMBO J.

[22] Li T, Kon N, Jiang L, Tan M, Ludwig T, Zhao Y (2012). Tumor suppression in the absence of p53-mediated cell-cycle arrest, apoptosis, and senescence. Cell.

[23] Kang MA, So EY, Simons AL, Spitz DR, Ouchi T (2012). DNA damage induces reactive oxygen species generation through the H2AX-Nox1/Rac1 pathway. Cell Death Dis.

[24] Farsinejad S, Gheisary Z, Ebrahimi Samani S, Alizadeh AM (2015). Mitochondrial targeted peptides for cancer therapy. Tumour Biol.

[25] Cheng AL, Kang YK, Chen Z, Tsao CJ, Qin S, Kim JS (2009). Efficacy and safety of sorafenib in patients in the Asia-Pacific region with advanced hepatocellular carcinoma: a phase III randomised, double-blind, placebo-controlled trial. Lancet Oncol.

[26] Azumi J, Tsubota T, Sakabe T, Shiota G (2016). miR-181a induces sorafenib resistance of hepatocellular carinoma cells through downregulation of RASSF1 expression. Cancer Sci.

[27] Zhu AX, Rosmorduc O, Evans TR, Ross PJ, Santoro A, Carrilho FJ (2015). SEARCH: a phase III, randomized, double-blind, placebo-controlled trial of sorafenib plus erlotinib in patients with advanced hepatocellular carcinoma. J Clin Oncol.

